# Gene Expression in the Aging Brain

**DOI:** 10.1371/journal.pbio.0030313

**Published:** 2005-08-02

No matter how healthy a life one leads, no person has managed to live much longer than a century. Even though the advances of the modern age may have extended the average human life span, it is clear there are genetic limits to longevity. One prominent theory of aging lays the blame on the accumulation of damage done to DNA and proteins by “free radicals,” highly reactive molecules produced by the metabolic activity of mitochondria. This damage is expected to reduce gene expression by damaging the DNA in which genes are encoded, and so the theory predicts that the most metabolically active tissues should show the greatest age-related reduction in gene expression. In this issue, Michael Eisen and colleagues show that the human brain follows this pattern. A similar pattern—which, surprisingly, involves different genes—is found in the brain of the aging chimpanzee.

The authors compared results from three separate studies of age-related gene expression, each done on the same type of DNA microarray and each comparing brain regions in young versus old adult humans. In four different regions of the cortex (the brain region responsible for higher functions such as thinking), they found a similar pattern of age-related change, characterized by changes in expression of hundreds of genes. In contrast, expression in one non-cortical region, the cerebellum (whose principal functions include movement), was largely unchanged with age. In addition to confirming a prediction of the free-radical theory of aging (namely, that the more metabolically active cortex should have a greater reduction in gene activity), this is the first demonstration that age-related gene expression patterns can differ in different cells of a single organism.

The authors found a similar difference in age-related patterns in the brain of the chimpanzee, with many genes down-regulated in the cortex that remained unchanged in the cerebellum. However, the set of affected cortical genes was entirely different between humans and chimps, whose lineages diverged about 5 million years ago. The explanation for this difference is unknown, but the finding highlights the fact that significant changes in gene expression patterns, and thus changes in many effects of the aging process, can accumulate over relatively short stretches of evolutionary time. [Fig pbio-0030313-g001]

**Figure pbio-0030313-g001:**
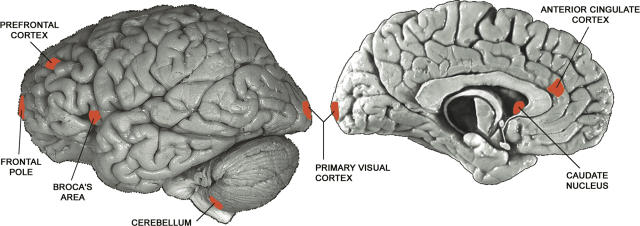
Gene expression data from three microarray studies of primate brains were used to identify genetic changes associated with aging in humans and chimps. Different brain regions in both primates undergo distinct age-related changes in gene expression

These results raise a number of questions about age-related gene expression changes, including whether metabolically active non-brain tissues display similar patterns of changes, and whether the divergence between human and chimp patterns was the direct result of selection, or was an inevitable consequence of some other difference in brain evolution. The patterns seen in this study also provide a starting point for understanding the network of genetic changes in aging, and may even reveal targets for treatment of neurodegenerative diseases.

